# Medical Progress

**Published:** 1894-02-24

**Authors:** 


### MEDICAL PROGRESS.

### DISEASES OF THE THROAT.

### Pharyngo-mycosis, Diphtheria, and Pseudo-diphtheria.

*Pharyngo-mycosis.*—This rare but troublesome affection is discussed at length by Dr. H. M. Thomas, of Chicago. Mycosis leptothrix occurs in two forms, diffuse and circumscribed. In the diffuse form the entire tongue is coated with a shiny, milk-white mass. In the circumscribed form, white, glistening points appear, resembling those seen in follicular tonsillitis, except that the mucous membrane about them preserves its normal pink hue. The tonsils, pharyngeal walls, and the base of the tongue are apt to be invaded. Some of these points resemble a mushroom in shape, others are like pointed cones. Dr. Fraenkel, of Berlin, first pointed out, in 1873, an abundance of leptothrix threads in these products.

From diphtheria this disease may be differentiated by the persistent discrete arrangement of the exudatæ, the general absence of surrounding inflammation, and the masses being harder than pseudo-membrane. The absence of the Klebs-Loeffler bacillus would be convincing. Mycosis of the pharynx may be confounded with cheesy masses in the crypts of the tonsil and follicular disease of the throat, but the ease with which the cheesy masses may be expressed from the crypts of the tonsil is in striking contrast to the fixation of the mycotic masses. The constant presence of inflammation in follicular disease, and the entire absence of inflammation in mycosis are distinctive of the two diseases.

*Symptoms.*—These, as the author states, are variable; sometimes there exists a sensation of tickling and dryness in the throat, confined to the pharynx. Again, there may be a feeling as if the throat had a band drawn around it; or slight pain on swallowing. Mycosis in the throat of a vocalist produces great dryness and irritability of the fauces after a short vocal use of the throat. In some cases the symptoms are greatly exaggerated, with depression and considerable fever.

*Treatment.*—The author has found that the galvano-cautery treatment for pharyngo-mycosis is the most nearly a specific in the management of this disease of anything we have. He uses a very fine elongated platinum point, which enables him to introduce it directly into each one of the crypts of the tonsil affected by the disease, and also to eradicate the punctated growth in other parts. At first he only makes three or four punctures at each treatment, which is undertaken three times a week. When the patient grows more tolerant of the irritation following the use of the galvano-cautery, the number of punctures at each treatment are increased. The bacillus thrives best in an acid medium; he therefore advises that "the negative pole be used for cauterisation, with a large positive pole, as the growth is thus subjected to a deeper action, and it is surrounded by an alkaline medium."

The author refers to the methods of treatment by swabbing with hyposulphate of sodium (Kitchen), peroxide of hydrogen, chlorate of potash gargle and tannic acid lozenges (Lemon), iodine and a gargle of borax (Leffert), alum and sulphur, bichloride of mercury gr. 1-2000 soln. as a gargle (Jacobson), but he discards all in favour of the very effectual galvano-cautery method.

*Diphtheria.*—The literature of diphtheria is too voluminous to give the gist of the various articles *seriatim*. The progress made in this affection consists in rendering more definite the large array of facts already in our possession.

*Etiology.*—That the immediate cause of diphtheria is the Klebs-Loeffler bacillus remains an undisputed fact. The lengthy correspondence in the *British Medical Journal* on the relation of diphtheria to drainage leaves little doubt that defective drainage is an efficient cause of the disease, that the relationship is more than the result of mere predisposition set up by sewer gas poisoning, and that Dr. Thorne Thorne has, in his recent work on diphtheria, "underestimated the influence of insanitary conditions as a cause of diphtheria" (Wilks)[2]. On the other hand we have the weighty expressions of Dr. Edward Seaton,[3] who states that "at schools, hospitals, and other institutions to which attention has been drawn by diphtheria prevalence, he knows of instances where the drainage has been altered at considerable expense first in one way then in another, and all apparently with no effect whatever on the incidence or recurrence of the disease. Indeed, it cannot be denied that some harm may have come from the belief in the all-sufficiency of drainage defects to account for the existence of diphtheria, which must tend to divert attention from precautions against infection."

*Pathology.*—It has long been recognised that scalds in the throat or the caustic action of such irritants as strong acids may produce a false membranous inflammation of the fauces and the larynx, but all other conditions in which a false membrane occurs have been classed as diptheritic; membranous croup has not been recognised as such, although some clinicians, *e.g.*, the late Hilton Fagge, have strenuously opposed the view that all membranous croups are really diphtheria. Kossel,[4] who has been recently investigating the bacteriology of diphtheria, states that it is generally believed now that the pseudo-diphtheria bacillus is only an attenuated form of the Klebs-Loeffler bacillus, that under proper conditions it may regain its virulence, and that the virulence of the diphtheria bacillus depends on (1) the alkalinity of the bouillon, (2) the age of the culture, (3) the size of the animal, (4) the site of the invasion. Dr. W. T. Councilman[5] endorses the view that the "pseudo-diphtheria bacillus" is simply a modified, less virulent form of diphtheritic bacillus, but reminds us that we cannot any longer maintain the prevalent view that all manner of inflammations of the pharynx in which a pseudo-membrane is present is *de facto* diphtheria. A fibrinous exudation, taking the form of a membrane which cannot be distinguished anatomically from true diphtheria, may be produced in a number of ways. It might, for instance, follow wounds and traumas of the pharynx, and in the air passages it may be easily produced by various irritants, as by the inhalation of the vapour of ammonia, or, as once occurred in his experience, from the accidental inhalation of croton oil which had been placed on the back of the tongue. A diphtheritic membrane may be produced in the rectum by the application of ammonia, or in various inflammations due to different bacteria. We regard now those only pseudo-membranous inflammations as belonging to diphtheria in which the Klebs-Loeffler bacillus is found. Like some other organisms, there is a marked variability in its virulence. Weigert and other investigators have shown that the diphtheritic membrane is due to a combination of necrosis and inflammation. The essential factor which determines the production of the membrane is necrosis of the surface epithelium. The necrotic tissue supplies the fibrin ferment, and fibrin is formed from the same exudation which comes in contact with the necrotic tissue; in whatever way necrotic tissue may be produced on a mucous surface, we shall have a certain amount of fibrinous exudation in and upon this; whether it be diphtheria or not depends on the presence or absence of the

*Characteristic Microbe.*—In man the action of the diptheritic bacilli is frequently complicated by the action of other organisms. There are other pathogenic organisms almost constantly present in the mouth, and of these, probably, the most important are the streptococcus pyogenes and the diplococcus lanceolatus. The staphylococcus aureus was found in his cultures less frequently than the streptococcus. It is uncertain whether the staphylococcus is capable in itself of exciting a fibrinous inflammation with the production of a membrane, but the fibrinous inflammations due to the streptococci are frequently found accompanying other infectious diseases, or they may be found alone. The streptococcus is certainly an active agent in producing most of the throat affections accompanying scarlet fever and measles, particularly the former. Although the appearance produced by the two organisms, the diphtheria bacillus and the streptococcus, may be so exactly similar that they cannot be distinguished from one another during the life of the individual, after death we can as a rule distinguish them.

After death the diphtheria bacillus is not found in the various organs of the body. Frosch has recently claimed that this view is incorrect, but Councilman adduces arguments against Frosch's results.

How may the diphtheria bacillus be detected during life for diagnostic purposes? Councilman employs two methods, one is by direct microscopical examination of the membrane. Small pieces of the membrane pulled off with forceps, or pledgets of sterilised cotton wool rubbed over the surface of the membrane, are afterwards rubbed over a cover glass. These are heated and stained with a Loeffler solution of methylene-blue, consisting of methylene-blue dissolved in a weak solution of caustic potash. But there is always a certain amount of difficulty in distinguishing diphtheritic bacilli from the other micro-organisms present, for we have no method of distinguishing them by staining, as in the case of tubercle bacilli.

As a culture medium Councilman recommends Loeffler's blood-serum and sugar bouillon. It consists of blood-serum obtained from the slaughter-house and mixed with one-fourth its volume of bouillon containing 1 per cent. of grape sugar. Ten or fifteen c.c. of this is poured into test tubes and sterilised in a hot-air chamber. The heat coagulates the albumen and renders the culture medium opaque. The pledget of cotton wool which has been rubbed over the membrane is then rubbed over the surface of the culture medium. The test tube is then placed in a warm chamber. At the end of twenty hours, if diphtheritic bacilli are present, an abundant growth will be found on the surface of the test tube medium. There is nothing characteristic of the diphtheritic bacilli in the microscopic appearances of the growth, but a microscopic examination of the cultures clears up all doubt.

A. W. W. Lea[6] recalls Sidney Martin's method. If any membrane can be seen remove a small piece with sterilised forceps, and place it rapidly in a sterilised blood-serum cultivating test-tube; if no membrane can be obtained, a little of the mucus from the throat may be taken and streaked in a test-tube with a platinum wire, previously sterilised by heat. The tube is put into an incubator, and kept at a temperature of 38 deg. C. If the bacilli are present, within twenty-four hours a typical growth will be obtained. The importance of such diagnostic methods is well illustrated by a case of pseudo-diphtheria or "false membrance of the throat," reported by Dr. George White.[7] The patient was under the care of Sir P. C. Smyly, and an examination revealed patches of yellowish membrane covering portions of both tonsils, uvula, and half the soft palate, but the membrane was easily removed by forceps without bleeding or reformation. Cultures showed the absence of this specific microbe.

Another suitable and readily-prepared culture medium is advocated by Schloffer,[8] who finds that by adding urine to agar broth we may obtain a nutrient medium which is thoroughly satisfactory. It consists of two parts of peptonised broth agar (2 per cent.) and one part of sterilised urine. This should first be subjected to a temperature of 70 deg. to 80 deg. C., and it may be improved as a culture medium by the addition of 6 per cent. of glycerine. The further procedures do not differ from those detailed above.

Further facts of clinical import are brought forward by F. H. Williams,[9] who has been working in association with Councilman. He has proved that true diphtheria may exist concurrently with scarlatina, measles, or typhoid fever, and also, what is equally important, that many cases admitted to the diphtheria wards with diphtheritic membrane were not diphtheria at all, thus demonstrating the necessity for bacteriological examinations, while in other cases of true diphtheria no membrane could be detected. On the other hand, Dr. Klein[10] has recently examined eight cases of typical diphtheria, "croups" of different forms, and of so-called "scarlatinal diphtheria." His examination of the scarlatinal cases fully confirmed the conclusions of Babes, Escherich, Holzinger, Wurtz, and others that, although simultaneous infection with diphtheria and scarlatina is possible, it may be laid down as a rule that diphtheritic symptoms appearing as a sequel to the fever—that is, one, two, or three weeks—are true diphtheria, and will yield the specific bacillus; but that the like symptoms occurring as a complication—that is, in the course of the fever—are not, and though the streptococcus of scarlatina may be present, Loeffler's bacillus will be looked for in vain.

Dr. Klein sums up his conclusions as follows: (1) Membranous croup of the larynx and trachea occurring concurrently with diphtheria of the fauces is true diphtheria. (2) The so-called scarlatinal diphtheria is not true diphtheria. (3) Diphtheria of the fauces and membranous croup, following sometimes after scarlatina, are both true diphtheria. (4) Membranous croup of the larynx and trachea, without diphtheria of the fauces, may be of two kinds: *a*, true diphtheria, or *b*, genuine fibrinous croup without the presence of the diphtheria bacillus, and therefore not true diphtheria.

*Treatment.*—F. H. Williams estimated the percentage of hæmoglobin in nineteen cases which appeared anæmic. In sixteen cases it was practically normal in amount, the other three were all children, and in these the percentage was 75, 50, and 50 per cent. It is doubtful, therefore, whether we need give iron in diphtheria for the purpose of combating the supposed anæmia, however useful its exhibition may be for other reasons. He advocates strong solutions of Peroxide of Hydrogen as a local germicide, either as a spray or swabbed over the affected parts.

The drawbacks of the strong solutions of hydrogen peroxide are (1) the strong solutions from their acidity cause some pain lasting one minute. We may disguise the acid by adding sugar. The amount of acid in Dr. Williams' 50 volume solution was only 1 per cent. (2) They are difficult to obtain. But a 10-volume solution has only to be evaporated over a water bath to obtain a solution of either about 25 or 50 volumes, by evaporating it to either one-third or one-sixth of its bulk respectively. (3) It is a bleaching agent, and must not be brought in contact with the hair or the skin.

The advantages claimed are: (1) The strong solution is a good germicide, yet non-poisonous, nor harmful to the mucous membrane; it cleanses a foul throat, and disintegrates certain portions of the membrane. (2) It assists in diagnosis, for even a weak solution causes any trace of membrane to assume a white colour. Speaking generally, the author advises the solution containing ½ per cent. of the acid should be applied every four hours. The 25-volume solution may be used as a spray; the 50-volume solution may be applied a drop or two at a time on a swab till the membrane is removed or much diminished. It is well to use cocaine before applying the peroxide. The author distinctly states that he does not believe that diphtheria can always be cured by even strong peroxide of hydrogen solutions, but he maintains that when properly used this is the most efficient and least harmful method he knows of.

Lea[11] recommends the application of a solution of perchloride of mercury in glycerine (1 in 500) for Loeffler has found that the perchloride is most effectual in destroying the bacilli. But the throat should first be cleansed by syringing with some antiseptic solution of a non-poisonous nature, such as tincture of iodine (1—40), Condy's fluid (1—40), or liquor sodæ chlorinatæ (1—20), and if there is any discharge from the nose this should be similarly syringed. The application of the perchloride solution should be made every three or four hours.

As there is good reason to believe that the bacilli may remain for some time after the membrane has disappeared, the applications should be continued, at longer intervals, of course, during convalescence.

"Acetous vapour" has been commended by W. A. Greet[12] for diphtheria, and he relates one desperate case in which it appeared to have been highly efficacious. The child was five years old, and the membrane had probably extended to the larynx. He had a quart cf malt vinegar put in a kettle on the fire, and the acetive vapour was steamed towards the child all night.

A spray of sulphurous acid, and the acid given internally in ♏x. doses in water every hour, is highly praised by Dr. Leeper.[13] He has found blowing sublimed sulphur constantly on to the fauces of absolutely no use whatever.

The antiseptic action of PHENIC ACID has been utilised by Sig. Bianchini Antonio[14] thus: Absorbent cotton, kept constantly moistened in a 2 per cent. solution, is worn about the neck, and it is carried to the affected parts by inspiration as it volatilises. Internally ten to thirty drops of tincture of chloride of iron are given hourly. In the more severe cases the affected parts are painted daily with the following: Salicylic acid, 3 parts; absolute alcohol, 20; resorcin, 2; and glycerin, 10 parts. The results claimed for this method are indeed encouraging.

PYOKTANIN in 3 per cent. solution applied three times daily is again claimed by C. Höring[15] to be a most effectual germicide in the local treatment of diphtheria. Simultaneously with the application the patients are syringed with lime water or use it as a gargle. Dr. L. Landes[16] describes a method used by Dr. Murison, of Cairo, a suggestion obtained from a Russian practitioner. His method is to take one part of EUCALYPTUS OIL and three parts of almond oil. giving one teaspoonful of the mixture every hour, Before the mixture is given a gargle of the pure oil is used if the child is old enough, and if not a spray instead, or both; and the shirt and pillow is also saturated with pure oil. If signs of intoxication occur reduce the dose to one-fourth. No results are given.

Kleb's ANTI-DIPHTHERIN has been tried by Kunne[17] in four cases of diphtheria at the Elberfeld Hospital. A 5 per cent. solution is applied three or four times daily. In two cases it was effectual, in the other two it had no effect.

[1] New York Med. Rec., Jan. 6, 1894. [2] Brit. Med. Jour., Dec. 16, 1893. [3] Ibid., Dec. 23, 1893. [4] Deut. Med. Woch., No. 46, 1893. [5] Am. Jour. of the Med. Sc., Nov., 1893. [6] The Hospital, Jan., 1894. [7] Jour. of Lar., Dec., 1893. [8] Central f. Bakt. Bd. XIV., No. 20. [9] Am. Journ. of the Med. Sc., Nov. 1893. [10] Wescit. [11] Am. Jonrn. of the Med. Sc., Dec., 1893. [12] Brit. Med. Jour., Jan. 27, 1894. [13] Ibid. [14] La Riforma Med., No. 204, 1893. [15] Memorabilien, Oct. 19, 1893. [16] Med. Rec., Dec. 16, 1893. [17] Wein. Med. Blätter, Dec. 14; Brit. Med. Jour., Dec. 30, 1893.

